# A systematic review of comparative studies of tiotropium Respimat® and tiotropium HandiHaler® in patients with chronic obstructive pulmonary disease: does inhaler choice matter?

**DOI:** 10.1186/s12890-016-0291-4

**Published:** 2016-10-11

**Authors:** Ronald Dahl, Alan Kaplan

**Affiliations:** 1 Odense University Hospital, Odense, Denmark; 2 Family Physician Airways Group of Canada, Richmond Hill, ON Canada

**Keywords:** Chronic obstructive pulmonary disease, Tiotropium Respimat®, Tiotropium HandiHaler®, Soft Mist™ Inhaler

## Abstract

**Background:**

In many countries worldwide, the long-acting anticholinergic drug tiotropium is available as a dry powder formulation delivered by means of the HandiHaler® inhalation device and as an aqueous solution delivered via the Respimat® Soft Mist™ Inhaler. Tiotropium HandiHaler® is a single-dose, dry powder, breath-actuated inhaler that provides delivered doses and lung deposition of tiotropium that are, over a wide range, not influenced by the severity of chronic obstructive pulmonary disease (COPD). Tiotropium Respimat® is a propellant-free, multi-dose inhaler that delivers a metered dose of medication as a fine, slow-moving, long-lasting soft mist, independently of patient inspiratory effort. The high fine-particle fraction of droplets produced by the Respimat® inhaler optimizes the efficiency of drug delivery to the lungs.

**Methods:**

To help inform the choice of tiotropium inhaler for prescribers and patients, this systematic review summarizes the available pharmacokinetic, efficacy and safety data from comparative studies of tiotropium Respimat® and tiotropium HandiHaler® in COPD, focusing on the licensed once-daily doses of 5 and 18 μg, respectively. Data sources reviewed include publications and abstracts identified from database searches.

**Results:**

Published evidence from comparative studies suggests that tiotropium Respimat® 5 μg and tiotropium HandiHaler® 18 μg provide similar clinical outcomes in patients with COPD.

**Conclusions:**

The findings indicate that physicians can base their decision about an inhaler for tiotropium on factors other than efficacy or safety. These could be patient preference for a particular inhaler, ease of use and the efficiency of drug delivery, with the aim of optimizing adherence and clinical outcomes with long-term tiotropium maintenance therapy.

Video (MP4 314368 kb)

**Electronic supplementary material:**

The online version of this article (doi:10.1186/s12890-016-0291-4) contains supplementary material, which is available to authorized users.

## Background

Current guidelines for the management of chronic obstructive pulmonary disease (COPD) recommend maintenance treatment with inhaled bronchodilator therapy and a variety of inhaler devices are currently available, with different technical properties, levels of drug deposition within the lungs and modes of operation. The choice of inhalation device is important because it can influence patients’ adherence to therapy, which can potentially affect long-term outcomes in a chronic disease such as COPD [1].

The long-acting anticholinergic drug tiotropium is available in many countries as a dry powder formulation delivered by means of the HandiHaler® inhaler device (Boehringer Ingelheim International GmbH, Ingelheim, Germany) [2, 3] and as an aqueous solution delivered via the Respimat® Soft Mist™ Inhaler (Boehringer Ingelheim International GmbH) [4, 5]. The efficacy and safety profile of tiotropium HandiHaler® in patients with COPD is well established, based on numerous clinical studies and also extensive post-marketing experience since its approval in Europe in 2002 and in the United States in 2004 [2–5]. Tiotropium Respimat® was approved as a COPD maintenance bronchodilator in 2007 in Europe and in 2014 in the United States and Canada [4–8].

Tiotropium HandiHaler® is a single-dose, dry powder, breath-actuated inhaler that provides consistent rates of delivered doses and lung deposition of tiotropium that are, over a wide range, not influenced by the severity of COPD [9]. In-vitro data have shown that the delivered dose of tiotropium was consistent at flow rates ranging from 20 to 60 L/min [10], and fine particle dose and fine particle fraction (defined as the mass fraction of particles with an aerodynamic diameter <5.8 μm) [11] were consistent at flow rates between 28.3 and 60 L/min, with a decline in fine particle dose of approximately 20% observed when flow rates decreased from 28.3 to 20 L/min [10]. In-vivo study data confirmed that COPD patients across a wide range of severities were able to generate sufficient inspiratory flow rates to activate the tiotropium HandiHaler® [10]. These findings indicate that the large majority of patients, irrespective of stage of COPD, can achieve acceptable delivery of medication through the tiotropium HandiHaler®.

Tiotropium Respimat® is a propellant-free multi-dose inhaler that uses mechanical power from a spring to deliver a metered dose of medication as a fine, slow-moving, long-lasting soft mist [11]. The inhaler was developed as an active system with a constant energy source, and the quality of dose and particle size distribution is uniquely independent of the patient’s inspiratory flow rate [12–15]. The tiotropium Respimat® inhaler aerosolizes the majority of each metered dose in the form of droplets of >1 μm (to avoid loss of small droplets during subsequent exhalation) and <5.8 μm (to facilitate efficient lung deposition through the mechanism of sedimentation); particles that are too large (≥6 μm) deposit in the oropharynx and large conducting airways, therefore having no clinical effect [11, 16, 17]. The fine particle fraction (defined as the proportion of drug mass in aerosolized particles that is carried by particles with an aerodynamic diameter of not more than 5.8 μm) is 65–80% [15, 18]. This high fine particle fraction, combined with the low velocity and long duration of the aerosol, results in a high level of drug deposition in the lungs and reduced oropharyngeal deposition [12, 14, 19]. This allows a more than 3-fold lower nominal dose of tiotropium to be administered compared with tiotropium HandiHaler® [7, 12, 20]; a quantitatively higher fraction of the inhaled dose is delivered to the bronchial system, with qualitatively higher distribution throughout the lung compared with other devices [21].

Both the tiotropium HandiHaler® and tiotropium Respimat® inhalers are available in many countries worldwide for the delivery of tiotropium as a maintenance treatment for COPD. Consequently, a detailed evaluation of their respective effects on clinical outcomes is warranted to help inform the choice of inhaler for prescribers and patients [7, 22]. The objective of this review was to summarize and evaluate the available pharmacokinetic, efficacy and safety data that have been published and presented to date from comparative studies of tiotropium Respimat® and tiotropium HandiHaler® in patients with COPD, focusing on the once-daily licensed doses of 5 and 18 μg, respectively.

## Methods

A systematic literature search was conducted for all interventional and non-interventional study publications containing the terms “tiotropium” AND “Respimat” AND “HandiHaler” AND “COPD”, using the following online sources: US National Library of Medicine, National Institutes of Health PubMed database; American Thoracic Society (ATS) and European Respiratory Society (ERS) congress abstracts; British Thoracic Society (BTS) congress abstracts (via the *Thorax* journal website) as well as the *Chest* journal website. Limits were not placed upon the language of the publication. The period searched was 2006–30 September 2015 for congress proceedings and any time up to 30 September 2015 for PubMed and the *Thorax* and *Chest* journal websites. Clinical trials were also searched, using the terms “tiotropium” AND “Respimat” AND “HandiHaler” AND “COPD” at www.clinicaltrials.gov.

The total hits from the search were assessed for their relevance (based on titles/abstracts), and those publications that were deemed potentially relevant (i.e. including comparative data for tiotropium Respimat® and tiotropium HandiHaler® in patients with COPD) were obtained in full for analysis. The researcher then examined the Methods and Results sections of the publications to extract and summarize the data for tiotropium Respimat® 5 μg and tiotropium HandiHaler® 18 μg.

Duplicate publications, republished papers, studies not comparing tiotropium Respimat® and tiotropium HandiHaler® efficacy, safety or pharmacokinetic data (at the licensed doses), studies in non-COPD patients and secondary/review publications that did not report original data were excluded from the analysis. The trial list from www.clinicaltrials.gov was compared against the literature search results to exclude trials with data already covered by the publications.

## Results

### Summary of search results

**Fig. 1:**
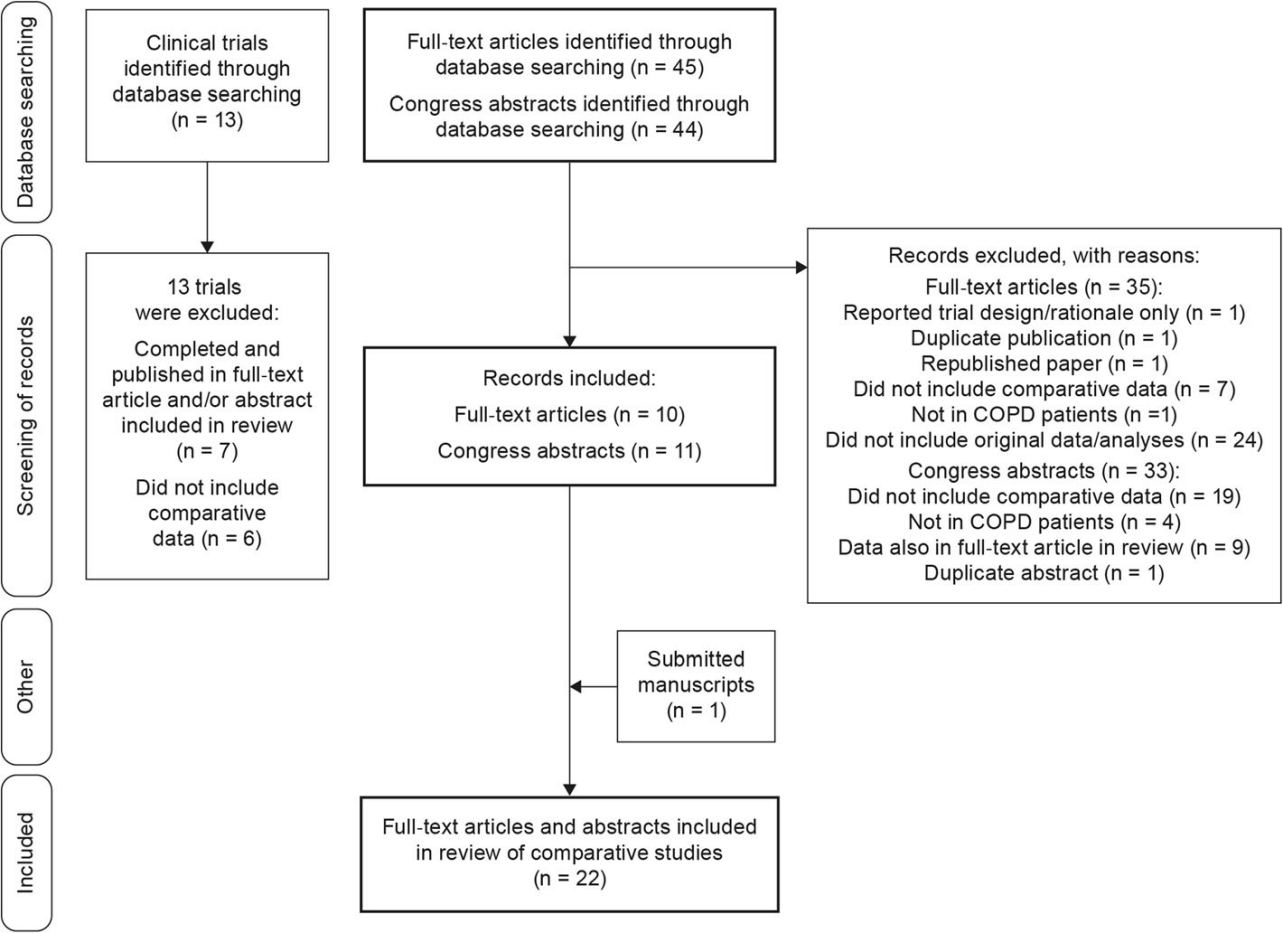
Flow diagram to show number of records identified, together with the numbers of records included and excluded, with reasons for exclusion

A total of 89 hits resulted from database searches (ATS abstracts = 10; ERS abstracts = 18; *Thorax* journal = 18 [including seven BTS abstracts and 11 other publications]; *Chest* journal = 13 [including nine meeting abstracts and four other publications]; PubMed = 30 publications). The number of records identified, included and excluded, and the reasons for exclusions are summarized in Fig. 1.

The total number of manuscript publications from this search that contained tiotropium Respimat® and tiotropium HandiHaler® data was 45. Of the 45 records, 35 publications were excluded for the following reasons: one reported trial design/rationale only (no results); one was a duplicate publication (included in both *Thorax* and PubMed search results); one was a republished paper (both publications were listed in the PubMed search results); seven studies did not include comparative efficacy, safety or pharmacokinetic data on tiotropium Respimat® and tiotropium HandiHaler® at the licensed doses; one study was not conducted in COPD patients; 24 publications did not include original data/analyses (13 review/commentary/opinion articles, eight correspondence articles, one editorial, one health care institute report; one treatment guidelines document). An additional manuscript submitted for publication was included in the analysis, as the authors considered it to be relevant (providing further evidence to address key questions posed in this review).

The number of congress presentation (ATS, ERS, BTS, *Chest*) abstracts from this search that contained tiotropium Respimat® and tiotropium HandiHaler® data was 44. Of the 44 records, 33 abstracts were excluded for the following reasons: 19 did not include comparative efficacy or safety data on tiotropium Respimat® and tiotropium HandiHaler® at the licensed doses; four studies were not in COPD; nine studies with data also published in full papers (full publications were listed in search results); and 1 was a duplicate abstract (presented at both BTS and ATS). Therefore, 22 publications in total were included in this review (11 manuscripts and 11 congress presentation abstracts) (for a complete list, see Additional file 1: Table S1) [23–44]. The characteristics of the clinical trials assessing tiotropium Respimat® and tiotropium HandiHaler® at the licensed doses (and reported as primary publications) and the pooled, combined and database studies covered by this review are summarized in Table 1.

**Table 1:** Primary tiotropium trials and pooled, combined and database analyses included in this review. Publications were limited to those reporting tiotropium Respimat® and tiotropium HandiHaler® data at the licensed doses

NCT identifier and citation(s)	Study design	COPD inclusion criteria	Baseline lung function values	Patient numbers and treatment groups	Endpoints
*Primary tiotropium trial publications*					
NCT02175342 Caillaud D, et al. Int J Chron Obstruct Pulmon Dis. 2007;2:559–65 [26]	Multicentre, randomized, double-blind within device (no blinding between RMT and HH), parallel-group, 3-week dose-ranging Phase II study	FEV_1_/FVC ≤70% FEV_1_ 30–65% predicted Smoking history ≥10 pack-years	Mean FEV_1_ 44% predicted	*n* = 202 RMT 5 μg (*n* = 25) HH 18 μg (*n* = 25)	Efficacy 1^o^: mean change in trough FEV_1_ from baseline to Day 21 2^o^: FVC and rescue medication use
NCT01222533 Hohlfeld JM, et al. J Clin Pharmacol. 2014;54:405–14 [31]	Comparative, multicentre, placebo-controlled, randomized (double-blind within RMT 1.25, 2.5, 5 μg; open-label HH 18 μg), 5-way crossover trial with 4-week treatment periods	FEV_1_/FVC <70% FEV_1_ < 80% predicted	FEV_1_/FVC 45% Mean FEV_1_ 54% predicted	*n* = 154 RMT 5 μg (*n* = 150) HH 18 μg (*n* = 146)	Efficacy 1^o^: trough FEV_1_ at end of 24-h dosing interval 2^o^: FVC, peak expiratory flow and rescue medication use
NCT00292448 Ichinose M, et al. Respir Med. 2010;104:228–36 [33]	Randomized, double-blind, double-dummy, 2-way, 4-week crossover, Phase II study of Japanese patients with COPD	FEV_1_/FVC ≤70% FEV_1_ ≤ 70% predicted Current or ex-smokers	FEV_1_/FVC 42% Mean FEV_1_ 43% predicted	*n* = 157 RMT 5 μg (*n* = 147) HH 18 μg (*n* = 147)	Efficacy 1^o^: trough FEV_1_ response 2^o^: peak and average FEV_1_ and FVC
NCT00239447 and NCT00281567 van Noord JA, et al. Respir Med. 2009;103:22–9 [37]	Pre-specified, pooled analysis of two identical, 30-week, double-blind, double-dummy, crossover studies (4-week crossover periods)	FEV_1_/FVC ≤70% FEV_1_ ≤ 60% predicted	Mean FEV_1_ 37% predicted	*n* = 207 Included in efficacy and safety analyses: RMT 5 μg (*n* = 189) HH 18 μg (*n* = 189)	Efficacy 1^o^: trough FEV_1_ from baseline to Day 29 2^o^: trough and peak FVC, FVC AUC_(0-12h),_ peak FEV_1_ and FEV_1_ AUC_(0-12h)_ at Day 29, and the time to therapeutic response
TIOSPIR® 205.452/NCT01126437 Wise RA, et al. N Engl J Med. 2013;369:1491–501 [48]	Randomized, double-blind, double-dummy, parallel-group, event-driven trial, duration 2–3 years	FEV_1_/FVC ≤70% FEV_1_ ≤ 70% predicted	Mean post-bronchodilator FEV_1_ 48% predicted for total population	*n* = 17,135 At risk, mortality RMT 5 μg (*n* = 5711) RMT 2.5 μg (*n* = 5730) HH 18 μg (*n* = 5694) At risk, exacerbation RMT 5 μg (*n* = 5705) RMT 2.5 μg (*n* = 5724) HH 18 μg (*n* = 5687)	Safety 1^o^: time to all-cause mortality Efficacy 1^o^: time to first COPD exacerbation Secondary outcomes: number of exacerbations; time to the first MACE
TIOSPIR® 205.452/NCT01126437 Anzueto A, et al. Respir Res. 2015;16:107 [24]	Spirometry sub-study Randomized, double-blind, double-dummy, parallel-group, event-driven trial, duration 2–3 years	FEV_1_/FVC ≤70% FEV_1_ ≤ 70% predicted	Mean post-bronchodilator FEV_1_ 48% predicted for total population	*n* = 1370 RMT 5 μg (*n* = 461) HH 18 μg (*n* = 445)	Trough FEV_1_ and FVC
Bouloukaki I, et al. Sleep Breath. 2015 [25, 44]	Randomized parallel-group trial	Mild to moderate COPD (resting arterial oxygen tension >60 mmHg while awake)	NR	*n* = 200 randomized RMT (*n* =100) HH (*n* =100 Patients analysed: RMT (*n* = 95) HH (*n* = 93)	SaO_2_ and sleep quality
*Pooled, combined and database analyses*					
Tashkin D, et al. Chest. 2014;146 r_Meeting Abstracts:49A [34]	16 clinical trials (13 tiotropium HandiHaler®, 3 tiotropium Respimat®)	Moderate to very severe COPD	NR	HH 18 μg (13 trials, *n* = 5646) Active comparator (2 trials, *n* = 584) Placebo (11 trials, *n* = 4853) RMT 5 μg (3 trials, *n* = 2219) RMT 10 μg (2 trials, *n* = 619) Placebo (3 trials, *n* = 2318)	HRQoL evaluated using the SGRQ
Dahl R, et al. Eur Respir J. 2014;44 Suppl 58:925 [28]	*Post-hoc*, pooled analysis of all placebo-controlled or head-to-head trials of RMT 5 μg and HH 18 μg with vital status follow up (analysed for death) and those with duration of at least 1 year (analysed for exacerbations)	COPD	NR	At risk of mortality, 6 trials: RMT 5 μg (*n* = 8760) HH 18 μg (*n* = 8680) Placebo (*n* = 6053) At risk of exacerbations, 5 trials: RMT 5 μg (*n* = 8314) HH 18 μg (*n* = 8673) Placebo (*n* = 5612)	Number of deaths Number of patients with ≥1 exacerbation
Halpin DMG, et al. Int J Chron Obstruct Pulmon Dis. 2015;10:239–59 [30]	Pooled analysis of adverse event data from 28 HH and 7 RMT studies	FEV_1_ ≤ 70% of FVC	Mean FEV_1_ 41% predicted	Patients treated: RMT 5 μg (*n* = 3282) RMT placebo (*n* = 3283) HH 18 μg (*n* = 9647) HH placebo (*n* = 8343)	Safety: AEs
Hohlfeld JM, et al. Int J Clin Pract. 2015;69:72–80 [32]	Combined analysis of all tiotropium trials in COPD involving Holter ECG monitoring and conducted between 2003 and 2012	FEV_1_ ≤ 70% of FVC	NR	4 trials (*n* = 727) HH 18 μg RMT 1.25–10 μg	Safety: incidence of cardiac arrhythmias
Tashkin D, et al. Eur Respir J. 2014;44 Suppl 58:923 [35]	Safety analysis in patients with renal impairment included in placebo-controlled trials of once-daily tiotropium Respimat® 5 μg (7 trials) or tiotropium HandiHaler® 18 μg (15 trials)	COPD and renal impairment	NR	*n* = 10,753 evaluable patients Normal renal function, mild and moderate renal impairment (respectively): HH 18 μg (*n* = 860), (n = 1099), (*n* = 448) HH placebo (*n* = 700), (n = 815), (*n* = 347) RMT 5 μg (*n* = 1104), (n = 1479), (*n* = 662) RMT placebo (*n* = 1040), (*n* = 1539), (*n* = 660)	Safety: AEs
Verhamme K, et al. Eur Respir J. 2013; 42 Suppl 57:4632 [38] Verhamme KM, et al. Eur Respir J. 2013;42: 606–15 [39]	Study of Integrated Primary Care Information Database (large Dutch primary care database)	COPD	NR	*n* = 11,287 (24,522 episodes of tiotropium use)	Safety: comorbidity

Abbreviations: *AE* adverse event, *AUC* area under the curve, *COPD* chronic obstructive pulmonary disease, *ECG* electrocardiogram, *FEV*_*1*_ forced expiratory volume in 1 s, *FVC* forced vital capacity, *HH* SPIRIVA® HandiHaler®, *HRQoL* health-related quality of life, *MACE* major adverse cardiovascular events, *NCT* National Clinical Trials database, *NR* not reported, *RMT* SPIRIVA®, Respimat®, *SaO*_*2*_ direct measurement of the oxygen content of the blood, *SGRQ* St George’s Respiratory Questionnaire, *TIOSPIR*® TIOtropium Safety and Performance In Respimat®

The search of www.clinicaltrials.gov provided 13 records and all 13 were excluded from the systematic review for the following reasons: seven trials were completed with data published in manuscripts or abstracts already selected for the current analysis; six trials did not include comparative data on tiotropium Respimat® and tiotropium HandiHaler® at the licensed doses (five trials compared tiotropium Respimat® or tiotropium HandiHaler® with other therapies [olodaterol or indacaterol]; one observational study showed only combined results for tiotropium Respimat® together with tiotropium HandiHaler®).

### Pharmacokinetic properties of tiotropium Respimat® and tiotropium HandiHaler®

In several clinical trials, tiotropium Respimat® 5 μg and tiotropium HandiHaler® 18 μg have demonstrated similar pharmacokinetic profiles [26, 31, 33, 37]. Urinary excretion (pre- and post-dose measures) of tiotropium Respimat® 5 μg was comparable with that of tiotropium HandiHaler® 18 μg [26] and plasma profiles were similar for tiotropium Respimat® 5 μg and tiotropium HandiHaler® 18 μg [33, 37].

**Fig. 2:**
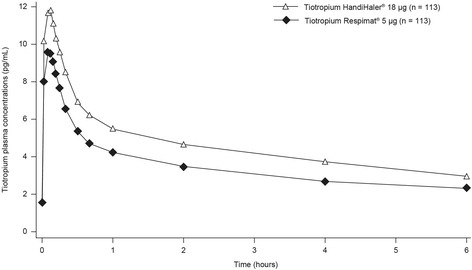
Geometric mean tiotropium plasma concentration–time profile following multiple inhalations using tiotropium Respimat® or tiotropium HandiHaler® [31]. (Adapted with permission from [31])

Previously, it had been suggested that systemic exposure with tiotropium Respimat® might be greater than with tiotropium HandiHaler®, with associated potential for increased risk of toxicity [45, 46]. However, a recent extensive study comparing the pharmacokinetic properties of tiotropium administered via the two inhalers showed that systemic exposure to tiotropium (as shown by mean plasma concentration profile at steady state) was lower in patients with COPD treated with tiotropium Respimat® 5 μg compared with patients treated with tiotropium HandiHaler® 18 μg [31]. The crossover design study included five 4-week treatment periods of placebo and once-daily doses of tiotropium Respimat® 1.25, 2.5 and 5 μg, and tiotropium HandiHaler® 18 μg. Based on the findings of earlier studies in COPD patients, which showed that pharmacokinetic steady state was achieved after 2–3 weeks of once-daily dosing with tiotropium, with no further accumulation after this time [37, 47], 4 weeks was considered to be sufficient to reach pharmacokinetic and pharmacodynamic steady state. Figure 2 shows mean plasma concentrations of tiotropium from 2 min to 6 h post-dosing with tiotropium HandiHaler® 18 μg and tiotropium Respimat® 5 μg.

### Efficacy of tiotropium Respimat® compared with tiotropium HandiHaler®

#### Lung function parameters

The efficacy of tiotropium Respimat® compared with tiotropium HandiHaler® has been compared in several short-term, randomized, double-blind, multicentre Respimat® dose-finding trials in patients with COPD [26, 31, 33, 37]. These studies showed that mean trough forced expiratory volume in 1 s (FEV_1_) and forced vital capacity (FVC) values after 3–4 weeks of treatment were most comparable for once-daily tiotropium Respimat® 5 μg and tiotropium HandiHaler® 18 μg (vs. alternative tiotropium Respimat® doses of 1.25, 2.5 or 10 μg).

#### Quality of life

**Fig. 3:**
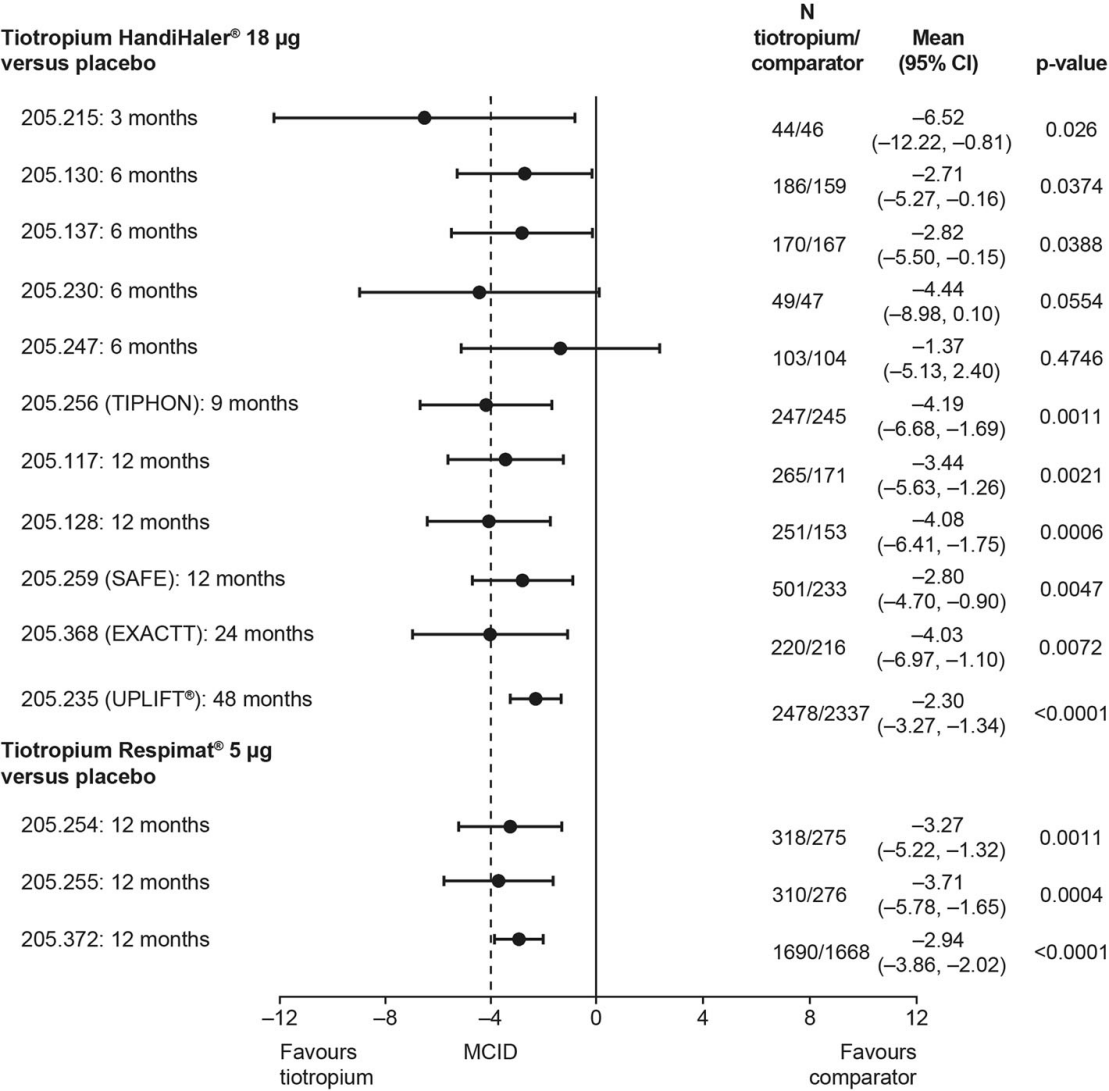
Forest plot of adjusted mean difference in SGRQ total score between tiotropium Respimat® or tiotropium HandiHaler® and placebo: results of a *post-hoc* pooled analysis [34]. CI, confidence interval; EXACTT, Exercise Endurance and COPD Treated With Tiotropium; MCID, minimal clinically important difference; SAFE, SPIRIVA® Assessment of FEV_1_; TIPHON, Tiotropium: Influence sur la Perception de l’amélioration des activités Habituelles Objectivée par une échelle Numérique; UPLIFT®, Understanding Potential Long-term Impacts on Function with Tiotropium

Data from 16 clinical trials (13 tiotropium HandiHaler®, three tiotropium Respimat®) were analysed to assess the effects of tiotropium, delivered via HandiHaler® or Respimat®, on health-related quality of life (HRQoL) in patients with moderate to very severe COPD (tiotropium HandiHaler® 18 μg, *n* = 5646; active comparator, two trials, *n* = 584; placebo comparator, 11 trials, *n* = 4853; tiotropium Respimat® 5 μg, three trials, *n* = 2219; tiotropium Respimat® 10 μg, two trials, *n* = 619; placebo comparator, three trials, *n* = 2318) [34]. HRQoL was evaluated using the St George’s Respiratory Questionnaire (SGRQ) total score. Although treatment effects varied slightly between trials, perhaps due to differences in study design, similar improvements in HRQoL were seen with tiotropium overall, irrespective of whether it was delivered by HandiHaler® or Respimat®. The mean change in SGRQ for tiotropium HandiHaler® compared with placebo ranged from –1.37 to –6.52 (statistically significant difference in 9 of 11 trials, *p* <0.05), and for tiotropium Respimat® 5 μg compared with placebo it ranged from –2.94 to –3.71 (statistically significant difference in all three trials, *p* <0.01) (Fig. 3) [34].

#### Sleep quality study

Patients with COPD can be affected by disordered gas exchange and poor sleep quality. A study was performed to compare the effect of tiotropium Respimat® and tiotropium HandiHaler® on sleeping arterial oxygen saturation (SaO_2_) and sleep quality in 200 patients with COPD, 6 months after the start of treatment [25, 44]. At the end of treatment (*n* = 188), both treatment groups showed significant improvements in minimum SaO_2_ (*p* <0.001) and percentage of sleep spent below 90% of SaO_2_ (TST90) (tiotropium Respimat®, *p* <0.001; tiotropium HandiHaler®, *p* = 0.002) compared with baseline (Respimat® vs. HandiHaler® for SaO_2_ and TST90 at 6 months: *p* = 0.83 and *p* = 0.04, respectively). Patients treated with tiotropium Respimat® had significantly better TST90 than did the patients treated with tiotropium HandiHaler®. Sleep disturbance was highly variable, but the durations of sleep stages (and therefore overall sleep quality) were significantly improved in the tiotropium Respimat® group compared with the tiotropium HandiHaler® group (*p* ≤0.01).

### Safety of tiotropium Respimat® compared with tiotropium HandiHaler®

In the Respimat*®* dose-finding trials [26, 31, 33, 37], tiotropium treatment was well tolerated compared with placebo, irrespective of the inhaler used, and similarly low numbers of patients using tiotropium Respimat® 5 μg and tiotropium HandiHaler® 18 μg reported adverse events.

#### Pooled and combined analyses of safety

A pre-specified pooled analysis of two 30-week crossover trials reported the following findings [37]: the most common adverse events were COPD exacerbations (9.6% with tiotropium Respimat® 5 μg, 11.2% with tiotropium HandiHaler® 18 μg and 13% with placebo) and nasopharyngitis (7.5% with tiotropium Respimat® 5 μg, 5.9% with tiotropium HandiHaler® 18 μg and 8.2% with placebo) [37]. COPD exacerbation, dry mouth and nasopharyngitis were also the most common adverse events in the study in Japanese patients, and the number of adverse events reported in patients receiving tiotropium Respimat® 5 μg and tiotropium HandiHaler® 18 μg was similar (45 [30.6%] and 41 [27.9%], respectively) [33].

A *post-hoc*, pooled, mixed-treatment analysis was performed of all placebo-controlled or head-to-head trials of tiotropium Respimat® 5 μg and tiotropium HandiHaler® 18 μg with vital status follow-up (analysed for death, *n* = 23,493), and those with duration of at least 1 year (analysed for exacerbations, *n* = 22,599) [28]. Tiotropium Respimat® 5 μg and tiotropium HandiHaler® 18 μg showed similar positive effects on mortality (odds ratio 1.01; 95% confidence interval [CI] 0.89–1.15) and exacerbations (odds ratio 0.90; 95% CI 0.81–1.01). Prolongation of survival was not statistically significant compared with placebo (odds ratios 0.92; 95% CI 0.77–1.10 for tiotropium Respimat® 5 μg and 0.91; 95% CI 0.80–1.04 for tiotropium HandiHaler® 18 μg). Risk of exacerbation was significantly lower for both tiotropium Respimat® 5 μg and tiotropium HandiHaler® 18 μg compared with placebo (odds ratios 0.79; 95% CI 0.70–0.88 and 0.87; 95% CI 0.78–0.98, respectively).

The safety of tiotropium delivered by Respimat® and HandiHaler® was recently reviewed in a pooled analysis of adverse event data from 28 HandiHaler® and seven Respimat® studies involving 12,929 patients treated with tiotropium and 11,626 patients treated with placebo [30]. Patients were eligible for inclusion in these studies if they had a diagnosis of COPD with FEV_1_ ≤ 70% of FVC, were aged ≥40 years and had ≥10 pack-years of smoking history. Patients were excluded if they had significant disease other than COPD. Other exclusion criteria in earlier studies were heart failure leading to hospitalization in the previous 3 years, cardiac arrhythmia requiring drug treatment or myocardial infarction (MI) within the past year. More recent trials only excluded life-threatening cardiac arrhythmia or arrhythmia that needed a change in medication or heart failure resulting in hospitalization in the past year, and/or MI within the previous 6 months. These relatively broad inclusion and exclusion criteria mean that the patient population included in the analysis reflected real-world heterogeneity of populations and phenotypes of COPD patients, as far as is possible in randomized clinical trials. The risk of adverse events (rate ratio 0.90; 95% CI 0.87–0.93) and serious adverse events (rate ratio (0.94; 95% CI 0.89–0.99) was significantly lower in the tiotropium group than in the placebo group, and the risk of fatal adverse events (rate ratio 0.90; 95% CI 0.79–1.01) and cardiac adverse events (rate ratio 0.93; 95% CI 0.85–1.02) was numerically lower in the tiotropium group. Similar results were obtained when tiotropium HandiHaler® 18 μg and tiotropium Respimat® 5 μg groups were analysed separately, and no increased risk of cardiac, vascular, and respiratory, thoracic and mediastinal disorders, or stroke, were observed in the tiotropium groups, except for a higher risk of ischaemic heart disease for tiotropium versus placebo in the tiotropium Respimat® 5 μg group (rate ratio 1.6 [95% CI 1.04–2.49]) but not in the tiotropium HandiHaler® 18 μg group. However, the incidence rates were lower in the placebo Respimat® group than in the placebo HandiHaler® group (1.25 vs. 1.89), and there was no evidence of increased risk of major adverse cardiovascular events (MACE) or fatal MACE in the tiotropium group compared with placebo, or in tiotropium HandiHaler® 18 μg and tiotropium Respimat® 5 μg groups separately. These results do not indicate an increased overall risk for fatal or cardiovascular events in COPD patients during tiotropium treatment, and support the findings from the TIOSPIR® (Tiotropium Safety and Performance in Respimat) trial—the largest randomized, double-blind, parallel-group study of patients with COPD, which did not show any relevant differences between tiotropium HandiHaler® 18 μg and tiotropium Respimat® 5 μg [48]. The findings of TIOSPIR® with regard to the safety of tiotropium HandiHaler® 18 μg and tiotropium Respimat® 5 μg are discussed in more detail later in this review.

The issue of whether inhaled anticholinergics and, in particular, tiotropium administered by Respimat®, may induce cardiac arrhythmias in a vulnerable subpopulation with cardiovascular morbidity has been discussed in the literature [39, 46, 49]. In this context, the results of a combined analysis of all tiotropium (HandiHaler® 18 μg and/or Respimat® 1.25–10 μg) trials in COPD involving Holter electrocardiogram (ECG) monitoring, and conducted between 2003 and 2012 [32], are important. In the four trials that were included in the analysis, patients were required to have a diagnosis of COPD with FEV_1_ ≤ 70% of FVC, were aged ≥40 years and had ≥10 pack-years of smoking history. Holter ECGs were evaluated for heart rate, pauses (absence of a heart beat for more than 3 seconds), supraventricular premature beats and ventricular premature beats. Maintenance therapy with either tiotropium Respimat® 5 μg or tiotropium HandiHaler® 18 μg was not associated with changes in any of these variables (Table 2). The authors commented that the results are in line with those of TIOSPIR®, which found no evidence that tiotropium Respimat® is associated with an increased risk of mortality, especially in patients with cardiac disease, or specifically arrhythmias at baseline.

**Table 2:** Summary of cardiac safety (Holter ECG) data for patients receiving tiotropium Respimat® or tiotropium HandiHaler® in four randomized trials. Placebo data are shown for comparison [32]

	Respimat® 5 μg	HandiHaler® 18 μg	Placebo
Average heart rate (BPM), mean ± SD (min–max)			
*Study 205.284*			
Baseline	–	79.89 ± 10.88 (59–108)	81.35 ± 9.14 (52–97)
Day 84	–	80.19 ± 9.78 (61–103)	81.12 ± 12.36 (54–140)
*Studies 205.254/255*			
Baseline	77.64 ± 10.05 (50–100)	–	79.26 ± 11.56 (55–136)
Day 281	77.23 ± 9.68 (56–99)	–	77.62 ± 11.21 (53–111)
*Study 205.458*			
Day 26	75.36 ± 10.77 (51–108)	75.83 ± 10.35 (58–100)	75.91 ± 10.91 (56–106)
Day 29	76.87 ± 10.82 (54–104)	77.39 ± 10.44 (55–104)	77.02 ± 10.36 (59–103)
Pauses, n/N (%)			
*Study 205.284*			
Baseline	–	2/74 (2.7)	1/65 (1.5)
Day 84	–	3/86 (3.5)	0
*Studies 205.254/255*			
Baseline	2/121 (1.7)	–	3/109 (2.8)
Day 281	1/103 (1.0)	–	2/73 (2.7)
*Study 205.458*			
Day 26	0	1/113 (0.9)	0
Day 29	0	0	0
VPB singles, n/N (%)			
*Study 205.284*			
Baseline	–	61/74 (82.4)	54/65 (83.1)
Day 84	–	71/86 (82.6)	58/78 (74.4)
*Studies 205.254/255*			
Baseline	112/121 (92.6)	–	95/109 (87.2)
Day 281	86/103 (83.5)	–	66/73 (90.4)
*Study 205.458*			
Day 26	90/112 (80.4)	88/113 (77.9)	93/117 (79.5)
Day 29	94/116 (81.0)	96/114 (84.2)	91/116 (78.4)
SVPB singles, n/N (%)			
*Study 205.284*			
Baseline	–	66/74 (89.2)	60/65 (92.3)
Day 84	–	82/86 (95.3)	74/78 (94.9)
*Studies 205.254/255*			
Baseline	113/121 (93.4)	–	101/109 (92.7)
Day 281	96/103 (93.2)	–	68/73 (93.2)
*Study 205.458*			
Day 26	100/112 (89.3)	105/113 (92.9)	111/117 (94.9)
Day 29	108/116 (93.1)	107/114 (93.9)	109/116 (94.0)

*BPM* beats per minute, *ECG* electrocardiogram, *FAS* full analysis set, *N* number of patients with non-missing data; n, number of patients with event, *SD* standard deviation, *SVPB* supraventricular premature beat, *VPB* ventricular premature beat. A pause was defined as absence of a heart beat for >3 s

**Fig. 4:**
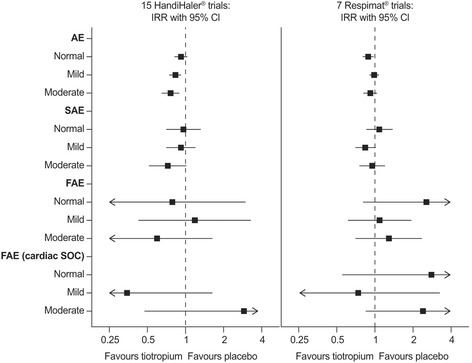
Forest plot of incidence rate ratios (95% CI) of on-treatment AEs by renal function at baseline: *post-hoc* analysis of tiotropium trials [35]. Renal function classification using National Institute for Health and Clinical Excellence (NICE) criteria: normal ≥90 mL/min, mild ≥60 to <90 mL/min, moderate ≥30 to <60 mL/min, severe <30 mL/min creatinine. Incidence rate ratios could not be calculated for severe renal impairment due to low patient numbers. Where there were no events in the placebo or tiotropium group, incidence rate ratios could not be calculated (division by zero) or are equal to zero, respectively, and are not graphically displayed. AE, adverse event; CI, confidence interval; FAE, fatal adverse event; IRR, incidence rate ratio; SAE, serious adverse event; SOC, System Organ Class (Medical Dictionary for Regulatory Activities)

An analysis of safety in patients with renal impairment (*n* = 10,753 evaluable patients) included in placebo-controlled trials of once-daily tiotropium Respimat® 5 μg (seven trials) or tiotropium HandiHaler® 18 μg (15 trials) has been conducted [35]. The incidence of adverse events, serious adverse events or fatal adverse events with either tiotropium Respimat® 5 μg or tiotropium HandiHaler® 18 μg showed no association with mild to moderately impaired renal function (Fig. 4). Results for severe renal impairment were limited due to the low number of patients (*n* = 52).

#### Database analysis of mortality

A report from a Dutch primary care database (source population 11,287, including 24,522 episodes of tiotropium use) found that the use of tiotropium Respimat® was associated with an almost 30% increase of mortality compared with tiotropium HandiHaler® [38, 39]. The association was strongest for cardiovascular/cerebrovascular death. These findings, however, are not supported by those of the large prospective TIOSPIR® trial, which showed no difference in mortality between patients using tiotropium Respimat® or tiotropium HandiHaler®, as described below.

### The TIOSPIR® study

The TIOSPIR® study was a 2–3 year, randomized, double-blind, parallel-group trial enrolling 17,135 patients with COPD [43, 48]. The aim of the trial was to evaluate the safety and efficacy of once-daily tiotropium Respimat® 2.5 or 5 μg and tiotropium HandiHaler® 18 μg in a large COPD population. Patients were permitted to continue their usual respiratory therapy (with the exception of other inhaled anticholinergics). Patients with cardiovascular diseases were allowed to participate, except for patients with heart failure resulting in hospitalization or cardiac arrhythmia requiring new drug treatment during the previous year, or experiencing MI within the past 6 months.

The primary safety endpoint for TIOSPIR® was time to all-cause mortality and the primary efficacy endpoint was time to first COPD exacerbation; secondary outcome measures included the number of exacerbations and time to the first MACE. For the primary endpoint of all-cause mortality, tiotropium Respimat® 5 μg was non-inferior to tiotropium HandiHaler® 18 μg (hazard ratio 0.96; 95% CI 0.84−1.09). Analysis of causes of death as assigned by TIOSPIR® investigators compared with those assigned by a mortality adjudication committee (MAC) found that fewer deaths were assigned by the MAC to cardiac disorders in the tiotropium HandiHaler® 18 μg group than in the tiotropium Respimat® 5 μg group, although this was not a significant effect (the CI of the rate ratio was overlapping 1) [42].

**Fig. 5:**
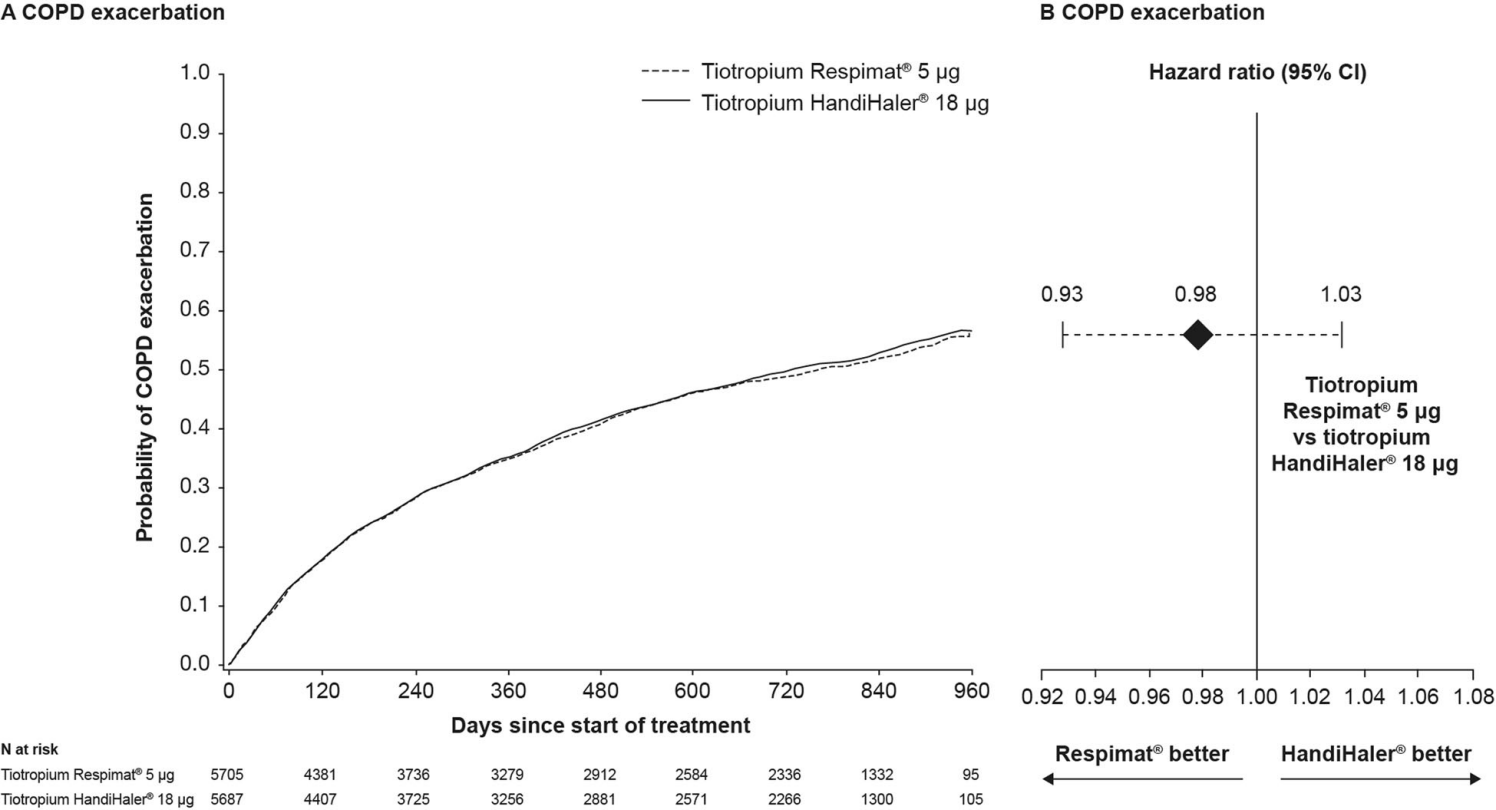
**a** Kaplan–Meier plot for COPD exacerbation in the TIOSPIR® trial. **b** Corresponding hazard ratio (95% confidence interval) for COPD exacerbation. (Adapted with permission from [48]). CI, confidence interval; COPD, chronic obstructive pulmonary disease

TIOSPIR® showed no significant difference between tiotropium Respimat® 5 μg and tiotropium HandiHaler® 18 μg for the primary efficacy endpoint of risk of first exacerbation (hazard ratio [HR] 0.98; 95% CI 0.93−1.03; *p* = 0.42). The proportions of patients with exacerbations (47.9% vs. 48.9%) and rates of exacerbations per patient-year (0.59; 95% CI 0.56−0.61 and 0.59; 95% CI 0.57−0.61) were similar between the tiotropium Respimat® 5 μg and tiotropium HandiHaler® 18 μg groups (Fig. 5).

Safety profiles of tiotropium Respimat® 5 μg and tiotropium HandiHaler® 18 μg were similar in the TIOSPIR® population. The incidence of MACE (3.9% vs. 3.6%) and causes of death (including death from cardiovascular causes, incidence 2.0% vs. 1.8%) were comparable for the tiotropium Respimat® 5 μg and tiotropium HandiHaler® 18 μg groups [48].

The spirometry sub-study of TIOSPIR® (*n* = 1370) found that tiotropium Respimat® 5 μg was non-inferior to tiotropium HandiHaler® 18 μg for adjusted mean trough FEV_1_ (averaged over 24–120 weeks: difference vs. HandiHaler® −10 mL; 95% CI −38−18) [23, 24, 48]. Adjusted mean trough FVC was also similar between treatment groups.

**Fig 6:**
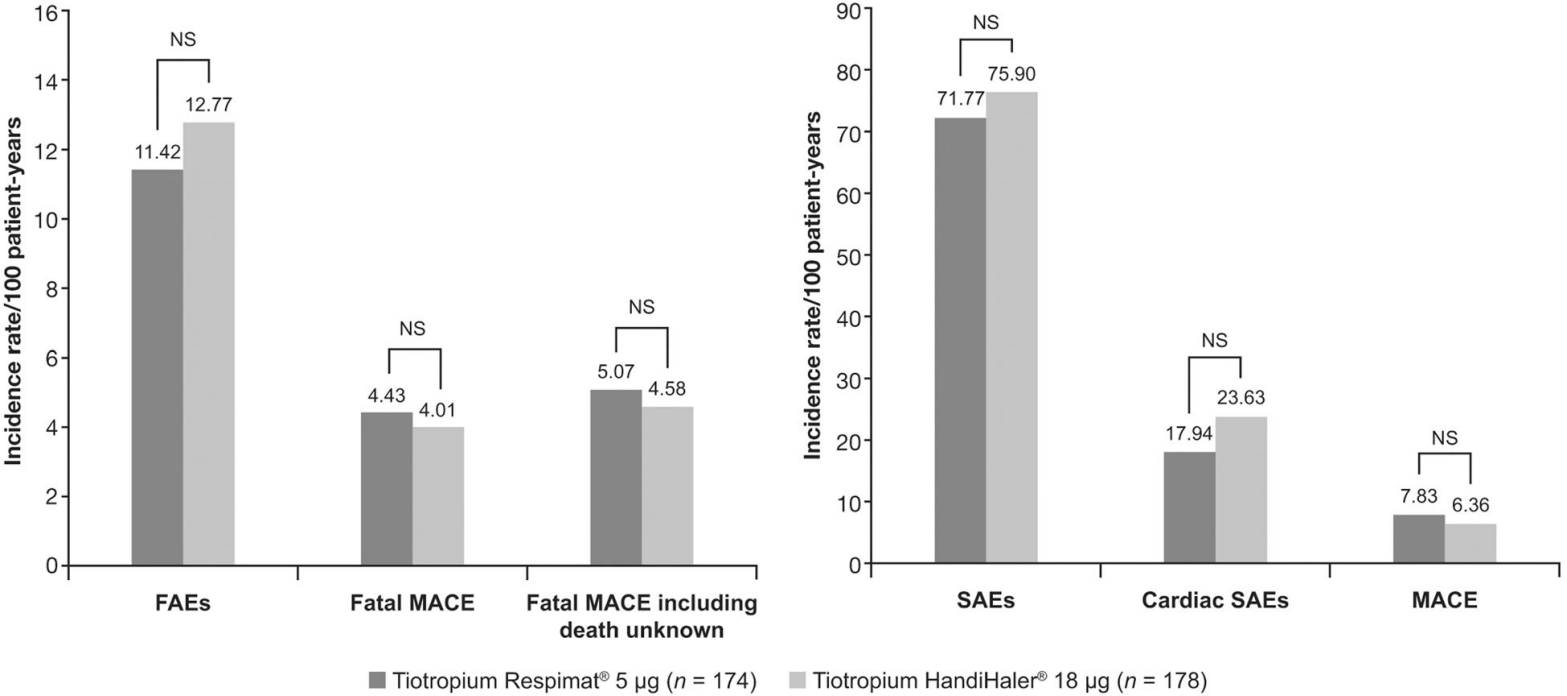
*Post-hoc* analysis of fatal, serious and cardiac AEs in patients receiving tiotropium Respimat® 5 μg and tiotropium HandiHaler® 18 μg and experiencing recent serious cardiac events during TIOSPIR® [41]. Events were counted from the day following the initial cardiac event through drug stop +30 days. FAEs, fatal adverse events; MACE, major adverse cardiovascular events; NS, not significant; SAEs, serious adverse events; TIOSPIR®, Tiotropium Safety and Performance in Respimat®

*Post-hoc* subgroup analyses including data from the TIOSPIR® study have further supported the clinical equivalence of tiotropium Respimat® 5 μg and tiotropium HandiHaler® 18 μg. Analyses of the 4-year placebo-controlled Understanding Potential Long-term Impacts on Function with Tiotropium (UPLIFT®) trial of tiotropium HandiHaler® and TIOSPIR® found that in patients who had experienced a cardiac event (for which they would have been excluded at baseline) during the trials, the risk of serious (including fatal) cardiac or MACE was not increased by tiotropium (tiotropium HandiHaler® 18 μg or tiotropium Respimat® 5 μg) [36]. Similar findings were obtained in a separate analysis of patients experiencing cardiac events during TIOSPIR® (Fig. 6) [41].

An analysis of data from patients from TIOSPIR® who were naïve to anticholinergic treatment at baseline (*n* = 6966) found that, as in the primary analysis [48], these patients had similar safety and exacerbation efficacy profiles when treated with tiotropium Respimat® 5 μg or tiotropium HandiHaler® 18 μg [40]. The HR was 0.93 (95% CI 0.75−1.17) for risk of death (measured as time to death) and 0.99 (95% CI 0.90−1.08) for exacerbations (measured as time to first exacerbation).

An analysis of patients treated with tiotropium HandiHaler® 18 μg at TIOSPIR® baseline, and who were randomized and subsequently received tiotropium Respimat® 5 μg during the study, showed that they had similar risks for all-cause mortality (HR 0.79; 95% CI 0.58–1.07), fatal MACE (HR 0.67; 95% CI 0.33–1.34), MACE (HR 0.69; 95% 0.44–1.08) and exacerbations (HR 0.96; 95% CI 0.86−1.08) as patients who continued to be treated with tiotropium HandiHaler® 18 μg [27, 29].

## Discussion

This systematic review evaluated pharmacokinetic, efficacy and safety results from published studies of tiotropium Respimat® and tiotropium HandiHaler® at the licensed doses (5 and 18 μg), respectively, with the aim of summarizing evidence that might inform the choice of tiotropium inhaler in clinical practice.

The results of several randomized dose-finding studies and the TIOSPIR® study have demonstrated that tiotropium Respimat® 5 μg has a pharmacokinetic, efficacy and safety profile that is comparable with that of tiotropium HandiHaler® 18 μg [24, 26, 31, 33, 37, 48]. Results from *post-hoc* and pooled analyses provide further confirmation that overall lung function, exacerbation, quality of life and safety outcomes are equivalent for the two tiotropium inhalers across a range of patient subtypes. The efficacy and safety of tiotropium, when administered by either Respimat® or HandiHaler®, is supported by previously published systematic reviews [7, 22, 49, 50].

There has been debate about the safety of inhaled anticholinergics and, in particular, the cardiac safety of tiotropium administered by Respimat®, which was triggered by meta-analysis [46] and database analysis [39] that reported an increase in mortality in patients treated with tiotropium Respimat®. The meta-analysis examined data from five randomized, controlled trials of tiotropium Respimat® [46] and found an increased risk of mortality compared with placebo. However, there was no direct comparison with tiotropium HandiHaler®, and the analysis was limited by differences in the populations studied, the tiotropium dose used, and length of follow-up. The investigators also noted that low event rates precluded precise estimates of risk [46]. The authors of the Dutch database analysis commented that it was unclear whether the apparent association between the use of tiotropium Respimat® and an increased risk of death was causal or due to residual confounding by COPD severity [39]. One source of confounding could be the substantial differences in the population treated with tiotropium HandiHaler® and tiotropium Respimat®. Although the analysis was adjusted for several factors, the adjustment was incomplete, and a channelling effect towards more severe patients being treated with tiotropium Respimat® was described by the same group [51].

The tiotropium safety data from the meta- and database analyses are in contrast to the results of TIOSPIR® [48], including ~17,000 patients, which provided the most robust data available to date regarding the comparative safety and efficacy of the two tiotropium formulations, and particularly the licensed doses. Key findings of TIOSPIR® were that tiotropium Respimat® 5 μg was non-inferior to tiotropium HandiHaler® 18 μg in terms of all-cause mortality and that the risk of cardiovascular mortality or MACE did not differ significantly between the two treatment groups [41, 43]. In addition, there was no increased risk of subsequent cardiac events with tiotropium HandiHaler® 18 μg or tiotropium Respimat® 5 μg in patients experiencing a serious cardiac event during the trial (the risk of MACE was actually lower with tiotropium HandiHaler® 18 μg than with placebo in UPLIFT®) [36]. This is an important finding, as many patients with COPD in clinical practice are likely to have underlying cardiac disease, yet such patients are typically excluded from randomized clinical trials of maintenance COPD treatments. The TIOSPIR® trial did not exclude most patients in routine care with cardiac diseases including stable coronary artery disease or stable arrhythmias, making it a study that was inclusive of the majority of patients that are typically seen in clinical practice. It is acknowledged that the data from TIOSPIR® are more robust than those arising from the meta-analyses or database studies [7, 52], which had previously raised concerns about an increased mortality risk with the tiotropium Respimat® inhaler [38, 39, 46].

Limitations of this review are the descriptive presentation of the findings (not subject to statistical analysis) and the inclusion of secondary and *post-hoc* analyses (such as those conducted on sub-populations of patients in the TIOSPIR® trial). Generally, it is challenging to draw firm conclusions from the results obtained across numerous trials, owing to differences in study duration and design. However, the studies assessed here included COPD patients across a broad range of disease severity (from moderate to very severe), and the TIOSPIR® trial allowed patients to receive tiotropium HandiHaler® or tiotropium Respimat® while continuing with their usual COPD maintenance therapy (thus helping to reflect clinical practice) [43, 48]. Overall, the review encompasses a large body of data on tiotropium HandiHaler® or tiotropium Respimat® from randomized trials, pooled analyses and database studies.

The studies reviewed here suggest that clinical efficacy appears equivalent between tiotropium Respimat® 5 μg and tiotropium HandiHaler® 18 μg, and as such, patient preferences and acceptance of different inhaler types become more important in the prescribing decision [53, 54]. Patient preference for an inhaler is an important determinant of treatment adherence, which is a key consideration for treatment choices in chronic diseases [1, 55, 56]. In addition, mishandling of inhalers is a common issue that may result in reduced symptom control [57], and therefore for any individual patient, it is important to assess ability to use the different types of available device. The inhalation and handling characteristics of tiotropium Respimat® have been assessed by patients with COPD, and was preferred to alternative inhalers, including metered-dose inhalers and dry powder inhalers [56, 58–60].

For physicians who may be considering whether patients are suitable candidates to switch from tiotropium HandiHaler® to tiotropium Respimat®, TIOSPIR® data show that in patients who switched from tiotropium HandiHaler® to tiotropium Respimat®, mortality, cardiac safety and exacerbation outcomes were similar to those who remained on tiotropium HandiHaler® [27, 29]. From the patient’s perspective, studies have suggested that they find it easy to switch from tiotropium HandiHaler® to tiotropium Respimat®, and have reported high levels of preference for, and adherence to, tiotropium Respimat® [61–65].

It also appears from “real-world” experience that physicians are already confident to prescribe tiotropium Respimat® for their patients with more severe disease and/or comorbidities. A study of the Dutch Integrated Primary Care Information Database was performed to compare patient characteristics at the time of the first prescription of tiotropium Respimat® or tiotropium HandiHaler® (source population 501,474, including 11,753 tiotropium users) [51]. COPD was found to be more severe and underlying comorbidities were more prevalent for first-time users of tiotropium Respimat® compared with tiotropium HandiHaler®. Also, in Italy, a drug utilization study conducted in patients receiving tiotropium during 2011–2012 found that users of tiotropium Respimat® and tiotropium HandiHaler® (*n* = 4390) had similar characteristics [66], but in this study, the probability of switching to tiotropium Respimat® was greater in patients with severe respiratory disease. If tiotropium Respimat® is being selected for patients with more severe COPD in clinical practice, this might help to explain the increased mortality risk suggested by earlier database and meta-analyses. However, TIOSPIR® demonstrated that tiotropium Respimat® has a similar safety and exacerbation efficacy profile to tiotropium HandiHaler® in patients with moderate-to-very severe COPD [48], supporting its use across the disease spectrum.

## Conclusions

The approval of tiotropium Respimat® in many countries has provided physicians with a choice of inhaler for the delivery of tiotropium maintenance therapy for their patients with COPD. Published evidence from comparative studies suggests that tiotropium Respimat® 5 μg and tiotropium HandiHaler® 18 μg provide similar clinical outcomes in COPD, indicating that physicians can choose between the two inhalers with confidence. Factors other than efficacy and safety, such as patient preference for a particular inhaler, ease of use and handling, and the efficiency of drug delivery (which has improved significantly for tiotropium with the Respimat® device) [20] should also be taken into account with the aim of optimizing adherence and clinical outcomes with long-term tiotropium maintenance therapy.

## Additional file

Additional file 1: Table S1. Including study details for all publications included in this review. (DOCX 48 kb)

## Abbreviations

ATS: American Thoracic Society
BTS: British Thoracic Society
CI: Confidence interval
COPD: Chronic obstructive pulmonary disease
ECG: Electrocardiogram
ERS: European Respiratory Society
FEV_1_: Forced expiratory volume in 1 s
FVC: Forced vital capacity
HR: Hazard ratio
HRQoL: Health-related quality of life
MAC: Mortality adjudication committee
MACE: Major adverse cardiovascular events
MI: Myocardial infarction
SaO_2_: Arterial oxygen saturation
SGRQ: St George’s Respiratory Questionnaire
TIOSPIR®: TIOtropium Safety and Performance In Respimat®
TST90: Percentage of sleep spent below 90% of SaO_2_
UPLIFT®: Understanding Potential Long-term Impacts on Function with Tiotropium.

## References

[1] Makela MJ, Backer V, Hedegaard M, Larsson K (2013). Adherence to inhaled therapies, health outcomes and costs in patients with asthma and COPD. Respir Med.

[2] SPIRIVA® HandiHaler® (tiotropium bromide inhalation powder) capsules for respiratory inhalation [prescribing information]. Ridgefield, CT, USA: Boehringer Ingelheim Pharmaceuticals Inc. 2014.

[3] Spiriva 18 microgram inhalation powder, hard capsule. SPC. Boehringer Ingelheim Ltd. http://www.medicines.org.uk/emc/medicine/10039/SPC/Spiriva+18microgram+inhalation+powder. Accessed 20 Aug 2015.

[4] SPIRIVA® Respimat® (tiotropium bromide) inhalation spray: for oral inhalation [prescribing information]. Ridgefield, CT, USA: Boehringer Ingelheim Pharmaceuticals, Inc. 2015.

[5] Spiriva (tiotropium) Respimat 2.5 micrograms, solution for inhalation. SPC. Boehringer Ingelheim Limited. https://www.medicines.org.uk/emc/medicine/20134. Accessed 20 Aug 2015.

[6] Product monograph SPIRIVA® Respimat® inhalation solution. Boehringer Ingelheim (Canada) Ltd. http://www.boehringer-ingelheim.ca/content/dam/internet/opu/ca_EN/documents/humanhealth/product_monograph/SpirivaRespimatPMEN.pdf. Accessed 20 Aug 2015.

[7] Keating GM (2014). Tiotropium Respimat(®) Soft Mist inhaler: a review of its use in chronic obstructive pulmonary disease. Drugs.

[8] Health Canada. ^Pr^STRIVERDI RESPIMAT. 17-6-2014.

[9] Brand P, Meyer T, Weuthen T, Timmer W, Berkel E, Wallenstein G (2007). Lung deposition of radiolabeled tiotropium in healthy subjects and patients with chronic obstructive pulmonary disease. J Clin Pharmacol.

[10] Chodosh S, Flanders JS, Kesten S, Serby CW, Hochrainer D, Witek TJJ (2001). Effective delivery of particles with the HandiHaler dry powder inhalation system over a range of chronic obstructive pulmonary disease severity. J Aerosol Med.

[11] Dalby RN, Eicher J, Zierenberg B (2011). Development of Respimat® Soft Mist Inhaler and its clinical utility in respiratory disorders. Med Devices (Auckl).

[12] Anderson P (2006). Use of Respimat Soft Mist inhaler in COPD patients. Int J Chron Obstruct Pulmon Dis.

[13] Hochrainer D, Holz H, Kreher C, Scaffidi L, Spallek M, Wachtel H (2005). Comparison of the aerosol velocity and spray duration of Respimat Soft Mist inhaler and pressurized metered dose inhalers. J Aerosol Med.

[14] Pitcairn G, Reader S, Pavia D, Newman S (2005). Deposition of corticosteroid aerosol in the human lung by Respimat Soft Mist inhaler compared to deposition by metered dose inhaler or by Turbuhaler dry powder inhaler. J Aerosol Med.

[15] Zierenberg B (1999). Optimizing the in vitro performance of Respimat. J Aerosol Med.

[16] Capstick TG, Clifton IJ (2012). Inhaler technique and training in people with chronic obstructive pulmonary disease and asthma. Expert Rev Respir Med.

[17] Lippmann M, Yeates DB, Albert RE (1980). Deposition, retention, and clearance of inhaled particles. Br J Ind Med.

[18] Dalby R, Spallek M, Voshaar T (2004). A review of the development of Respimat Soft Mist Inhaler. Int J Pharm.

[19] Newman SP, Steed KP, Reader SJ, Hooper G, Zierenberg B (1996). Efficient delivery to the lungs of flunisolide aerosol from a new portable hand-held multidose nebulizer. J Pharm Sci.

[20] Ninane V, Vandevoorde J, Cataldo D, Derom E, Liistro G, Munghen E (2015). New developments in inhaler devices within pharmaceutical companies: A systematic review of the impact on clinical outcomes and patient preferences. Respir Med.

[21] Ciciliani A-M, Wachtel H, Langguth P. Comparing Respimat® Soft Mist™ inhaler and DPI aerosol deposition by combined in vitro measurements and CFD simulations**.** Presented at: Respiratory Drug Delivery 2014; 4-8 May 2014; Fajardo, Puerto Rico.

[22] Keating GM (2012). Tiotropium bromide inhalation powder: a review of its use in the management of chronic obstructive pulmonary disease. Drugs.

[23] Anzueto A, Wise R, Pledger G, Calverley P, Dusser D, Cotton D (2013). The Tiotropium Safety and Performance in Respimat (TIOSPIR) trial: bronchodilator efficacy in a spirometry substudy [abstract]. Chest.

[24] Anzueto A, Wise R, Calverley P, Dusser D, Tang W, Metzdorf N (2015). The Tiotropium Safety and Performance in Respimat® (TIOSPIR®) Trial: Spirometry Outcomes. Respir Res.

[25] Bouloukaki I, Giannadaki K, Merigkis C, Michelakis S, Mauroudi E, Moniaki V (2014). Tiotropium Respimat versus HandiHaler to improve sleeping oxygen saturation and sleep quality in COPD [abstract]. Eur Respir J.

[26] Caillaud D, Le Merre C, Martinat Y, Aguilaniu B, Pavia D (2007). A dose-ranging study of tiotropium delivered via Respimat Soft Mist Inhaler or HandiHaler in COPD patients. Int J Chron Obstruct Pulmon Dis.

[27] Calverley P, Anzueto A, Dahl R, Meuller A, Fowler A, Metzdorf N, et al. Tiotropium Safety and Performance In Respimat® (tiospir™): safety and efficacy in patients with tiotropium Handihaler® use at baseline [abstract]. Thorax. 2014;69 Suppl 2:A192 (P262).

[28] Dahl R, Schmidt H, Könen-Bergmann M, Metzdorf N (2014). Mixed treatment analysis comparing tiotropium HandiHaler® and Respimat® [abstract]. Eur Respir J.

[29] Dahl R, Calverley P, Anzueto A (2015). Safety and efficacy of tiotropium in patients switching from HandiHaler® to Respimat® in the TIOSPIR® trial. Submitted manuscript. BMJ Open.

[30] Halpin DM, Dahl R, Hallmann C, Mueller A, Tashkin D (2015). Tiotropium HandiHaler(®) and Respimat(®) in COPD: a pooled safety analysis. Int J Chron Obstruct Pulmon Dis.

[31] Hohlfeld JM, Sharma A, van Noord JA, Cornelissen PJ, Derom E, Towse L (2014). Pharmacokinetics and pharmacodynamics of tiotropium solution and tiotropium powder in chronic obstructive pulmonary disease. J Clin Pharmacol.

[32] Hohlfeld JM, Furtwaengler A, Konen-Bergmann M, Wallenstein G, Walter B, Bateman ED (2015). Cardiac safety of tiotropium in patients with COPD: a combined analysis of Holter-ECG data from four randomised clinical trials. Int J Clin Pract.

[33] Ichinose M, Fujimoto T, Fukuchi Y (2010). Tiotropium 5microg via Respimat and 18microg via HandiHaler; efficacy and safety in Japanese COPD patients. Respir Med.

[34] Tashkin D, Jones P, Leonard T, Liu D, Metzdorf N, Zubeck V, Wise R. Tiotropium delivered via HandiHaler or Respimat: improvement in health-related quality of life in patients with chronic obstructive pulmonary disease [abstract]. Chest Journal. 2014;146(4_MeetingAbstracts):49A.

[35] Tashkin D, Metzdorf N, Hallmann C, Konen-Bergmann M, Kupas K, Dalby R (2014). Safety of tiotropium in renally impaired patients [abstract]. Eur Respir J.

[36] Tashkin D, Kowey PR, Fowler A, Metzdorf N, Dewberry H, Mueller A (2015). Cardiac safety of tiotropium in patients with cardiac events: a retrospective, combined analysis of the UPLIFT® and TIOSPIR™ trials [abstract]. Am J Respir Crit Care Med.

[37] van Noord JA, Cornelissen PJ, Aumann JL, Platz J, Mueller A, Fogarty C (2009). The efficacy of tiotropium administered via Respimat Soft Mist Inhaler or HandiHaler in COPD patients. Respir Med.

[38] Verhamme K, van Blijderveen N, Romio S, Stricker B, Brusselle G, Sturkenboom M (2013). Chronic kidney disease as effect modifier in the association between the use of tiotropium Respimat and mortality [abstract]. Eur Respir J.

[39] Verhamme KM, Afonso A, Romio S, Stricker BC, Brusselle GG, Sturkenboom MC (2013). Use of tiotropium Respimat Soft Mist Inhaler versus HandiHaler and mortality in patients with COPD. Eur Respir J.

[40] Wise R, Calverley P, Dahl R, Dusser D, Metzdorf N, Mueller A, et al. Tiotropium safety and performance in respimat® (tiospir™): safety and efficacy in patients naïve to treatment with anticholinergics [abstract]. Thorax. 2014;69 Suppl 2:A192 (P261).

[41] Wise R, Fowler A, Metzdorf N, Dewberry H, Mueller A, Kowey PR. Safety of tiotropium in patients with cardiac events in the TIOSPIR® trial**.** Presented at: European Respiratory Society International Congress; September 26-30, 2015; 26 September 2015.

[42] Wise R, Kowey PR, Austen G, Lawton A, Mueller A, Metzdorf N, et al. Investigator-reported versus adjudicated cause of death in the TIOSPIR® trial. Presented at: European Respiratory Society International Congress; September 26-30, 2015; 26 September 2015.

[43] Wise RA, Anzueto A, Calverley P, Dahl R, Dusser D, Pledger G (2013). The Tiotropium Safety and Performance in Respimat Trial (TIOSPIR), a large scale, randomized, controlled, parallel-group trial-design and rationale. Respir Res.

[44] Bouloukaki I, Tzanakis N, Mermigkis C, Giannadaki K, Moniaki V, Mauroudi E (2015). Tiotropium Respimat Soft Mist Inhaler versus HandiHaler to improve sleeping oxygen saturation and sleep quality in COPD. Sleep Breath.

[45] Beasley R (2013). Tiotropium Respimat increases the risk of mortality: pro. Eur Respir J.

[46] Singh S, Loke YK, Enright PL, Furberg CD (2011). Mortality associated with tiotropium mist inhaler in patients with chronic obstructive pulmonary disease: systematic review and meta-analysis of randomised controlled trials. BMJ.

[47] Durham MC (2004). Tiotropium (Spiriva): a once-daily inhaled anticholinergic medication for chronic obstructive pulmonary disease. Proc (Bayl Univ Med Cent).

[48] Wise RA, Anzueto A, Cotton D, Dahl R, Devins T, Disse B (2013). Tiotropium Respimat inhaler and the risk of death in COPD. N Engl J Med.

[49] Karner C, Chong J, Poole P (2014). Tiotropium versus placebo for chronic obstructive pulmonary disease. Cochrane Database Syst Rev.

[50] Kaplan A (2010). Effect of tiotropium on quality of life in COPD: a systematic review. Prim Care Respir J.

[51] Verhamme KMC, Afonso AS, Romio S, Stricker BC, Brusselle GG, Sturkenboom MC (2012). Patient characteristics at first time use of tiotropium Handihaler vs tiotropium Respimat: is there a potential of channelling? [abstract]. Am J Respir Crit Care Med.

[52] Barnes NC, Jones PW, Davis KJ (2014). Safety of tiotropium through the Handihaler: why did meta-analyses and database studies appear to give a false alarm?. Thorax.

[53] Dekhuijzen PN, Vincken W, Virchow JC, Roche N, Agusti A, Lavorini F (2013). Prescription of inhalers in asthma and COPD: towards a rational, rapid and effective approach. Respir Med.

[54] Vincken W, Dekhuijzen PR, Barnes P (2010). ADMIT Group. The ADMIT series - Issues in inhalation therapy. 4) How to choose inhaler devices for the treatment of COPD. Prim Care Respir J.

[55] Borel JC, Pepin JL, Pison C, Vesin A, Gonzalez-Bermejo J, Court-Fortune I, et al. Long-term adherence with non-invasive ventilation improves prognosis in obese COPD patients. Respirology. 2014;19:857–65. 10.1111/resp.12327 24912564

[56] Hodder R, Price D (2009). Patient preferences for inhaler devices in chronic obstructive pulmonary disease: experience with Respimat Soft Mist inhaler. Int J Chron Obstruct Pulmon Dis.

[57] Melani AS, Bonavia M, Cilenti V, Cinti C, Lodi M, Martucci P (2011). Inhaler mishandling remains common in real life and is associated with reduced disease control. Respir Med.

[58] Freytag F, Rau-Berger H, Glaab T, Wolf K (2007). Respimat® Soft Mist™ inhaler preferred to Diskus by patients with COPD and/or asthma [abstract]. Am J Respir Crit Care Med.

[59] Hodder R, Reese PR, Slaton T (2009). Asthma patients prefer Respimat Soft Mist Inhaler to Turbuhaler. Int J Chron Obstruct Pulmon Dis.

[60] Schurmann W, Schmidtmann S, Moroni P, Massey D, Qidan M (2005). Respimat Soft Mist inhaler versus hydrofluoroalkane metered dose inhaler: patient preference and satisfaction. Treat Respir Med.

[61] ADESPI Adherence to Spiriva® in Patients With COPD (Chronic Obstructive Pulmonary Disease), Measured by Morisky-8 (MMAS-8) Scale, in Routine Medical Practice. NCT01388166. https://www.clinicaltrials.gov/ct2/show/NCT01388166. Accessed 20 Aug 2015.

[62] Asakura Y, Nishimura N, Maezawa K, Terajima T, Kizu J, Chohnabayashi N (2013). Effect of switching tiotropium HandiHaler® to Respimat® Soft Mist™ Inhaler in patients with COPD: the difference of adverse events and usability between inhaler devices. J Aerosol Med Pulm Drug Deliv.

[63] Hanada S, Wada S, Ohno T, Sawaguchi H, Muraki M, Tohda Y (2015). Questionnaire on switching from the tiotropium HandiHaler to the Respimat inhaler in patients with chronic obstructive pulmonary disease: changes in handling and preferences immediately and several years after the switch. Int J Chron Obstruct Pulmon Dis.

[64] Nishimura N, Asakura Y, Junta T, Suda R, Yamao S, Tomishima Y (2014). Therapeutic effect of switching tiotropium HandiHaler to Respimat Soft Mist™ inhaler in the patients with COPD: the difference of adverse events and adherence to manipulations between inhaler devices [abstract]. Am J Respir Crit Care Med.

[65] Suzuki S, Sagara H, Tanaka A, Yamaguchi M, Ohta S, Homma T (2014). Effect of switching administration of tiotropium, HandiHaler to Respimat Soft Mist Inhaler in COPD patients: focus on physical characteristics and background of COPD patients before changing inhaler devices. Am J Respir Crit Care Med.

[66] Trotta F, Da Cas R, Rajevic M, Rossi M, Traversa G (2015). Risk factors influencing the prescription of tiotropium Respimat formulation: a population-based cohort study. BMJ Open.

