# Supplementary material for: A systematic review of comparative studies of tiotropium Respimat® and tiotropium HandiHaler® in patients with chronic obstructive pulmonary disease: does inhaler choice matter?
Source: BMC Pulm Med. 2016 Oct 11;16:135. doi: 10.1186/s12890-016-0291-4 (PMC5057252; doi:10.1186/s12890-016-0291-4)
Supplement: Additional file 1: Table S1. — Including study details for all publications included in this review. (DOCX 48 kb) [file 12890_2016_291_MOESM1_ESM.docx]

**Additional File 1 Table 1** Study details for all publications included in this review.

Publications were limited to those reporting tiotropium Respimat^®^ and tiotropium HandiHaler^®^ data at the licensed doses.

| **NCT identifier and citation(s)** | **Study design** | **COPD inclusion criteria** | **Baseline lung function values** | **Patient numbers and treatment groups**  **(randomized unless specified otherwise)** | **Endpoints** |
| --- | --- | --- | --- | --- | --- |
| ***Primary studies*** | | | | | |
| **NCT02175342**  Caillaud D, et al. Int J Chron Obstruct Pulmon Dis. 2007;2:559–65 [1] | Multicentre, randomized, double-blind within device (no blinding between RMT and HH), parallel-group, 3-week dose-ranging Phase II study | FEV_1_/FVC ≤70%  FEV_1_ 30–65% predicted  Smoking history ≥10 pack-years | Mean FEV_1_ 44% predicted | Total n = 202  RMT 1.25 µg n = 25  RMT 2.5 µg n = 28  RMT 5 µg n = 25  RMT 10 µg n = 26  RMT 20 µg n = 26  Placebo n = 24  HH 18 µg n = 25  HH placebo n = 23 | Efficacy 1^o^: mean change in trough FEV_1_ from baseline to Day 21  2^o^: FVC and rescue medication use |
| **NCT01222533**  Hohlfeld JM, et al. J Clin Pharmacol. 2014;54:405–14 [2] | Comparative, multicentre, placebo-controlled, randomized (double-blind within RMT 1.25, 2.5, 5 μg; open-label HH 18 µg), 5-way crossover trial with 4-week treatment periods | FEV_1_/FVC <70%  FEV_1_ ≤80% predicted | FEV_1_/FVC 45%  Mean FEV_1_ 54% predicted | Total randomized: n = 154 Treated set:  RMT 5 µg n = 150  RMT 2.5 µg n = 145  RMT 1.25 µg n = 147  HH 18 µg n = 146  Placebo n = 147 | Efficacy 1^o^: trough FEV_1_ at end of 24-h dosing interval  2^o^: FVC, peak expiratory flow and rescue medication use |
| **NCT00292448**  Ichinose M, et al. Respir Med. 2010;104:228–36 [3] | Randomized, double-blind, double-dummy, 2-way, 4-week crossover, Phase II study of Japanese patients with COPD | FEV_1_/FVC ≤70%  FEV_1_ ≤70% predicted  Current or ex-smokers | FEV_1_/FVC 42%  Mean FEV_1_ 43% predicted | Total randomized: n = 157 Treated set:  RMT 5 µg n = 147  HH 18 µg n = 147 | Efficacy 1^o^: trough FEV_1_ response  2^o^: peak and average FEV_1_ and FVC |
| **TIOSPIR^®^ 205.452/NCT01126437**  Wise RA, et al. N Engl J Med. 2013;369:1491–501 [4] | Randomized, double-blind, double-dummy, parallel-group, event-driven trial, duration 2–3 years | FEV_1_/FVC ≤70%  FEV_1_ ≤70% predicted | Mean post-bronchodilator FEV_1_ 48% predicted for total population | Total n = 17,135  At risk, mortality  RMT 5 µg n = 5711  RMT 2.5 µg n = 5730  HH 18 µg n = 5694  At risk, exacerbation  RMT 5 µg n = 5705  RMT 2.5 µg n = 5724  HH 18 µg n = 5687 | Safety 1^o^: time to all-cause mortality  Efficacy 1^o^: time to first COPD exacerbation  Secondary outcomes: number of exacerbations; time to the first MACE |
| **TIOSPIR^®^ 205.452/NCT01126437**  Anzueto A, et al. Respir Res. 2015;16:107 [5] | Randomized, double-blind, double-dummy, parallel-group, event-driven trial, duration 2–3 years | FEV_1_/FVC ≤70%  FEV_1_ ≤70% predicted | Mean post-bronchodilator FEV_1_ 48% predicted for total population | Treated set:  Total n = 1370 (spirometry sub-study)  RMT 5 µg n = 461  RMT 2.5 µg n = 464  HH 18 µg n = 445 | Trough FEV_1_ and FVC |
| **TIOSPIR^®^ 205.452/NCT01126437**  Wise R, et al. ERS 2015. Poster PA1498 [6] | Randomized, double-blind, double-dummy, parallel-group, event-driven trial, duration 2–3 years | FEV_1_/FVC ≤70%  FEV_1_ ≤70% predicted  Patients who died during the study | Mean post-bronchodilator FEV_1_ 48% predicted for total population | Patients analysed:  Total n = 1302  RMT 5 µg n = 423  RMT 2.5 µg n = 440  HH 18 µg n = 439 | Safety: causes of death (MAC-adjudicated vs.  investigator-reported) |
| **TIOSPIR^®^ 205.452/NCT01126437**  Wise R, et al. ERS 2015. Poster PA985 [7] | Randomized, double-blind, double-dummy, parallel-group, event-driven trial, duration 2–3 years | FEV_1_/FVC ≤70%  FEV_1_ ≤70% predicted  Patients experiencing an initial cardiac event during the trial | Mean post-bronchodilator FEV_1_ 48% predicted for total population | Patients analysed:  Total n = 442  RMT 5 µg n = 221  HH 18 µg n = 221 | Safety: all-cause mortality, serious cardiac SAEs, MACE |
| **TIOSPIR^®^ 205.452/NCT01126437**  Dahl R, et al. Manuscript submitted to BMJ Open [8] | Switching from HH to RMT in TIOSPIR**^®^** 205.452  Randomized, double-blind, double-dummy, parallel-group, event-driven trial, duration 2–3 years | FEV_1_/FVC ≤70%  FEV_1_ ≤70% predicted  Patients switching from HH to RMT | Mean post-bronchodilator FEV_1_ 48% predicted for total population | Treated patients:  Total n = 2784  RMT 5 µg n = 918  RMT 2.5 µg n = 914  HH 18 µg n = 952 | Safety 1^o^: time to all-cause mortality  Efficacy 1^o^: time to first COPD exacerbation  Secondary outcomes: number of exacerbations; time to the first MACE |
| **NCT00239447 and NCT00281567**  van Noord JA, et al. Respir Med. 2009;103:22–29 [9] | Pre-specified, pooled analysis of 2 identical, 30-week, double-blind, double-dummy, crossover studies (4-week crossover periods) | FEV_1_/FVC ≤70%  FEV_1_ ≤60% predicted | Mean FEV_1_ 37% predicted | Total randomized: n = 207  Included in efficacy and safety analyses:  RMT 5 µg n = 189  RMT 10 µg n = 185  HH 18 µg n = 189  Placebo n = 186 | Efficacy 1^o^: trough FEV_1_ from baseline to Day 29  2^o^: trough and peak FVC, FVC AUC_(0-12h),_ peak FEV_1_ and FEV_1_ AUC_(0-12h)_ at  Day 29 and the time to therapeutic response |
| Bouloukaki I, et al. Sleep Breath. 2015; E-pub before print [10] | Randomized parallel-group trial | Mild to moderate COPD (resting arterial oxygen tension >60 mmHg while awake) | NR | Total randomized: n = 200  RMT n = 100  HH n = 100  Patients analysed:  RMT n = 95  HH n = 93 | SaO_2_ and sleep quality |
| ***Post-hoc analysis*** | | | | | |
| Halpin DMG, et al. Int J Chron Obstruct Pulmon Dis. 2015;10:239–59 [11] | Pooled analysis of adverse event data from 28 HH and 7 RMT studies | FEV_1_ ≤70% of FVC | Mean FEV_1_ 41% predicted | Patients treated:  RMT 5 µg n = 3282  RMT placebo n = 3283  HH 18 µg n = 9647  HH placebo n = 8343 | Safety: AEs |
| Hohlfeld JM, et al. Int J Clin Pract. 2015;69:72–80 [12] | Combined analysis of all tiotropium trials in COPD involving Holter ECG monitoring, and conducted between 2003 and 2012 | FEV_1_ ≤70% of FVC |  | Four trials, total n = 727  HH 18 µg  RMT 1.25–10 µg | Safety: incidence of cardiac arrhythmias |
| Verhamme KM, et al. Eur Respir J. 2013;42:606–15 [13] | Study of Integrated Primary Care Information database (large Dutch primary care database) | COPD | NR | Source population, total n = 11,287  Episodes of tiotropium use, total n = 24,522 | Safety: all-cause mortality |
| ***Abstracts*** | | | | | |
| Anzueto A, et al. Chest. 2013;144 4 Meeting Abstracts:1027A [14] | Spirometry sub-study of TIOSPIR**^®^** 205.452.  Randomized, double-blind, double-dummy, parallel-group, event-driven trial, duration 2–3 years | FEV_1_/FVC ≤70%  FEV_1_ ≤70% predicted | Mean post-bronchodilator FEV_1_ 48% predicted for total population | Total n = 1370  RMT 5 µg n = 461  RMT 2.5 µg n = 464  HH 18 µg n = 445 | Trough FEV_1_ and FVC |
| Bouloukaki I, et al. Eur Respir J. 2014;44 Suppl 58: P3280 [15] | Randomized parallel-group trial | Mild to moderate COPD (resting arterial oxygen tension >60 mmHg while awake) | NR | Total randomized: n = 200  RMT n = 100  HH n = 100  Patients analysed:  Total n = 188  RMT n = 95  HH n = 93 | SaO_2_ and sleep quality |
| Calverley P, et al. Thorax. 2014;69 Suppl 2:A192 [16] | Patients switching from HH to RMT in TIOSPIR**^®^** 205.452  Randomized, double-blind, double-dummy, parallel-group, event-driven trial, duration 2–3 years | FEV_1_/FVC ≤70%  FEV_1_ ≤70% predicted  Patients switching from HH to RMT | Mean post-bronchodilator FEV_1_ 48% predicted for total population | Patients analysed:  Total n = 1779  RMT 5 µg n = 602  RMT 2.5 µg n = 572  HH 18 µg n = 605 | Safety 1^o^: time to all-cause mortality  Efficacy 1^o^: time to first COPD exacerbation  Secondary outcomes: number of exacerbations; time to the first MACE |
| Dahl R, et al. Eur Respir J. 2014;44 Suppl 58:925 [17] | *Post-hoc*, pooled analysis of all placebo-controlled or head-to-head trials of RMT 5 µg and HH 18 µg with vital status follow up (analyzed for death) and those with duration of at least 1 year (analyzed for exacerbations) | COPD | NR | At risk analysis of mortality, 6 trials:  RMT 5 µg n = 8760  HH 18 µg n = 8680  Placebo n = 6053  At risk analysis of exacerbations, 5 trials:  RMT 5 µg n = 8314  HH 18 µg n = 8673  Placebo n = 5612 | Number of deaths  Number of patients with ≥1 exacerbation |
| Tashkin D, et al. Chest. 2014;146 r_Meeting Abstracts:49A [18] | 16 clinical trials (13 tiotropium HandiHaler^®^, 3 tiotropium Respimat^®^) | Moderate to very severe COPD | NR | HH 18 µg (13 trials, n = 5646)  Active comparator (2 trials, n = 584)  Placebo (11 trials, n = 4853)  RMT 5 µg (3 trials, n = 2219)  RMT 10 µg (2 trials, n = 619)  Placebo (3 trials, n = 2318) | HRQoL evaluated using the SGRQ |
| Tashkin D, et al. Eur Respir J*.* 2014;44 Suppl 58:923 [19] | Safety analysis in patients with renal impairment included in placebo-controlled trials of once-daily tiotropium Respimat^®^ 5 μg (7 trials) or tiotropium HandiHaler^®^ 18 μg (15 trials) | COPD and renal impairment | NR | Total n = 10,753 evaluable patients  Normal renal function, mild and moderate renal impairment (respectively):  HH 18 µg n = 860, n = 1099, n = 448  HH placebo n = 700, n = 815, n = 347  RMT 5 µg n = 1104, n = 1479, n = 662  RMT placebo n = 1040, n = 1539, n = 660 | Safety: AEs |
| Tashkin DP, et al. Am J Respir Crit Care Med. 2015;191:A5770. Poster presented at ATS 2015 [20] | Analysis of cardiac safety in patients experiencing cardiac events during UPLIFT^®^ and TIOSPIR^®^ | FEV_1_/FVC ≤70%  FEV_1_ ≤70% predicted | NR | UPLIFT^®^:  HH 18 μg n = 353  Placebo n = 376  TIOSPIR^®^:  RMT 5 μg n = 174  HH 18 μg n = 178 | Cardiac safety |
| Verhamme K, et al. Eur Respir J. 2013;42 Suppl 57:4632 [21] | Study of Integrated Primary Care Information Database (large Dutch primary care database) | COPD | NR | Source population, total n = 11,287  Episodes of tiotropium use, total n = 24,522 | Safety: comorbidity |
| Wise R, et al. Thorax. 2014; 69 Suppl 2:A192 [22] | Sub-study of TIOSPIR**^®^** 205.452.  Randomized, double-blind, double-dummy, parallel-group, event-driven trial, duration 2–3 years | FEV_1_/FVC ≤70%  FEV_1_ ≤70% predicted  Patients naive to anticholinergics | Mean post-bronchodilator FEV_1_ 48% predicted for total population | Patients analysed:  Total n = 6966  RMT 5 µg n = 2312  RMT 2.5 µg n = 2345  HH 18 µg n = 2309 | Safety 1^o^: time to all-cause mortality  Efficacy 1^o^: time to first COPD exacerbation  Secondary outcomes: number of exacerbations; time to the first MACE |

Abbreviations: AE, adverse event; AUC, area under the curve; COPD, chronic obstructive pulmonary disease; ECG, electrocardiogram; FEV_1_, forced expiratory volume in 1 second; FVC, forced vital capacity; HH, tiotropium HandiHaler^®^; HRQoL, health-related quality of life; MAC, mortality adjudication committee; MACE, major adverse cardiovascular event; NCT, National Clinical Trials database; NR, not reported; RMT, tiotropium Respimat^®^; SaO_2_, direct measurement of the oxygen content of the blood; SAE, serious adverse event; SGRQ, St George’s Respiratory Questionnaire; TIOSPIR^®^, TIOtropium Safety and Performance In Respimat^®^; UPLIFT^®^, Understanding Potential Long-term Impacts on Function with Tiotropium

**References**

1. Caillaud D, Le Merre C, Martinat Y, Aguilaniu B, Pavia D. A dose-ranging study of tiotropium delivered via Respimat Soft Mist Inhaler or HandiHaler in COPD patients. Int J Chron Obstruct Pulmon Dis. 2007;2:559-65.

2. Hohlfeld JM, Sharma A, van Noord JA, Cornelissen PJ, Derom E, Towse L, et al. Pharmacokinetics and pharmacodynamics of tiotropium solution and tiotropium powder in chronic obstructive pulmonary disease. J Clin Pharmacol. 2014;54:405-14.

3. Ichinose M, Fujimoto T, Fukuchi Y. Tiotropium 5microg via Respimat and 18microg via HandiHaler; efficacy and safety in Japanese COPD patients. Respir Med. 2010;104:228-36.

4. Wise RA, Anzueto A, Cotton D, Dahl R, Devins T, Disse B, et al. Tiotropium Respimat inhaler and the risk of death in COPD. N Engl J Med. 2013;369:1491-501.

5. Anzueto A, Wise R, Calverley P, Dusser D, Tang W, Metzdorf N, et al. The Tiotropium Safety and Performance in Respimat(R) (TIOSPIR(R)) Trial: Spirometry Outcomes. Respir Res. 2015;16:107.

6. Wise R, Kowey PR, Austen G, Lawton A, Mueller A, Metzdorf N, et al. Investigator-reported versus adjudicated cause of death in the TIOSPIR^®^ trial**.** Presented at: European Respiratory Society International Congress; September 26-30, 2015; 26 September 2015.

7. Wise R, Fowler A, Metzdorf N, Dewberry H, Mueller A, Kowey PR. Safety of tiotropium in patients with cardiac events in the TIOSPIRâ trial**.** Presented at: European Respiratory Society International Congress; September 26-30, 2015; 26 September 2015.

8. Dahl R, Calverley P, Anzueto A. Safety and efficacy of tiotropium in patients switching from HandiHaler® to Respimat® in the TIOSPIR^®^ trial. Submitted manuscript. BMJ Open. 2015.

9. van Noord JA, Cornelissen PJ, Aumann JL, Platz J, Mueller A, Fogarty C. The efficacy of tiotropium administered via Respimat Soft Mist Inhaler or HandiHaler in COPD patients. Respir Med. 2009;103:22-9.

10. Bouloukaki I, Tzanakis N, Mermigkis C, Giannadaki K, Moniaki V, Mauroudi E, et al. Tiotropium Respimat Soft Mist Inhaler versus HandiHaler to improve sleeping oxygen saturation and sleep quality in COPD. Sleep Breath. 2015.

11. Halpin DM, Dahl R, Hallmann C, Mueller A, Tashkin D. Tiotropium HandiHaler(^®^) and Respimat(^®^) in COPD: a pooled safety analysis. Int J Chron Obstruct Pulmon Dis. 2015;10:239-59.

12. Hohlfeld JM, Furtwaengler A, Konen-Bergmann M, Wallenstein G, Walter B, Bateman ED. Cardiac safety of tiotropium in patients with COPD: a combined analysis of Holter-ECG data from four randomised clinical trials. Int J Clin Pract. 2015;69:72-80.

13. Verhamme KM, Afonso A, Romio S, Stricker BC, Brusselle GG, Sturkenboom MC. Use of tiotropium Respimat Soft Mist Inhaler versus HandiHaler and mortality in patients with COPD. Eur Respir J. 2013;42:606-15.

14. Anzueto A, Wise R, Pledger G, Calverley P, Dusser D, Cotton D, et al. The Tiotropium Safety and Performance in Respimat (TIOSPIR) trial: bronchodilator efficacy in a spirometry substudy [abstract]. Chest. 2013;144 4_Meeting Abstracts:1027A.

15. Bouloukaki I, Giannadaki K, Merigkis C, Michelakis S, Mauroudi E, Moniaki V, et al. Tiotropium Respimat versus HandiHaler to improve sleeping oxygen saturation and sleep quality in COPD [abstract]. Eur Respir J. 2014;44 Suppl 58:P3280.

16. Calverley P, Anzueto A, Dahl R, Mueller A, Fowler A, Metzdorf N, et al. Tiotropium Safety and Performance In Respimat^®^ (TioSPIR™): safety and efficacy in patients with tiotropium Handihaler^®^ use at baseline [abstract]. Thorax. 2014;69 Suppl 2:A192.

17. Dahl R, Schmidt H, Könen-Bergmann M, Metzdorf N. Mixed treatment analysis comparing tiotropium HandiHaler^®^ and Respimat^®^ [abstract]. Eur Respir J. 2014;44 Suppl 58:925.

18. Tashkin D, Jones P, Leonard T, Liu D, Metzdorf N, Zubeck V, et al. Tiotropium delivered via HandiHaler or Respimat: improvement in health-related quality of life in patients with chronic obstructive pulmonary disease [abstract]. Chest. 2014;146 r_Meeting Abstracts:49A.

19. Tashkin D, Metzdorf N, Hallmann C, Konen-Bergmann M, Kupas K, Dalby R. Safety of tiotropium in renally impaired patients [abstract]. Eur Respir J. 2014;44 Suppl 58:923.

20. Tashkin D, Kowey PR, Fowler A, Metzdorf N, Dewberry H, Mueller A, et al. Cardiac safety of tiotropium in patients with cardiac events: a retrospective, combined analysis of the UPLIFT^®^ and TIOSPIR™ trials [abstract]. Am J Respir Crit Care Med. 2015;191:A5770. Poster presented at ATS 2015.

21. Verhamme K, van Blijderveen N, Romio S, Stricker B, Brusselle G, Sturkenboom M. Chronic kidney disease as effect modifier in the association between the use of tiotropium Respimat and mortality [abstract]. Eur Respir J. 2013;42 Suppl 57:4632.

22. Wise R, Calverley P, Dahl R, Dusser D, Metzdorf N, Mueller A, et al. Tiotropium Safety and Performance In Respimat^®^ (TioSPIR™): safety and efficacy in patients naïve to treatment with anticholinergics [abstract]. Thorax. 2014;69 Suppl 2:A192.
